# High rates of posttraumatic stress symptoms in women living with HIV in Canada

**DOI:** 10.1371/journal.pone.0200526

**Published:** 2018-07-19

**Authors:** Anne C. Wagner, Denise Jaworsky, Carmen H. Logie, Tracey Conway, Neora Pick, Denise Wozniak, Jesleen Rana, Wangari Tharao, Angela Kaida, Alexandra de Pokomandy, Allyson Ion, Lori A. Chambers, Kath Webster, S. Jay MacGillivray, Mona Loutfy, on behalf of the CHIWOS Research Team

**Affiliations:** 1 Department of Psychology, Ryerson University, Toronto, Ontario, Canada; 2 Faculty of Medicine, University of British Columbia, Vancouver, British Columbia, Canada; 3 Factor-Inwentash Faculty of Social Work, University of Toronto, Toronto, Ontario, Canada; 4 Women’s College Research Institute, Women’s College Hospital, Toronto, Ontario, Canada; 5 Oak Tree Clinic, BC Women’s Hospital, Vancouver, British Columbia, Canada; 6 CHIWOS Community Advisory Board, Mission, British Columbia, Canada; 7 Women’s Health in Women’s Hands Community Health Centre, Toronto, Ontario, Canada; 8 Department of Medicine, University of Toronto, Toronto, Ontario, Canada; 9 Faculty of Health Sciences, Simon Fraser University, Burnaby, British Columbia, Canada; 10 Chronic Viral Illness Service, McGill University Health Centre, Montreal, Quebec, Canada; 11 Department of Family Medicine, McGill University, Montreal, Quebec, Canada; 12 School of Social Work, McMaster University, Hamilton, Ontario, Canada; 13 St. Michael’s Hospital, Toronto, Ontario, Canada; Stellenbosch University, SOUTH AFRICA

## Abstract

**Purpose:**

Women living with HIV experience high levels of trauma exposure before and after diagnosis. One of the most challenging outcomes following trauma exposure is posttraumatic stress disorder. Despite high exposure to traumatic events, the presence and contributors to posttraumatic stress disorder symptoms have not been examined in women living with HIV in Canada.

**Methods:**

The current study examines the presence of, contributors to, and geographical regions associated with self-reported posttraumatic stress symptoms (PTSS) among 1405 women enrolled in the Canadian HIV Women’s Sexual & Reproductive Health Cohort Study (CHIWOS).

**Results:**

Separate linear regression models were run for the three provinces in the cohort: British Columbia, Ontario and Québec. Scores consistent with posttraumatic stress disorder were reported by 55.9%, 39.1% and 54.1% of the participants in each province, respectively (*F*(2, 1402) = 13.53, *p* < .001).

**Conclusions:**

The results demonstrate that women living with HIV have high rates of PTSS, and that rates and variables associated with these symptoms vary by province. These results suggest the need for trauma-informed practices and care for women living with HIV in Canada, which may need to be tailored for the community and identities of the women.

## Introduction

Women account for approximately 25% of new cases of HIV in Canada [1]. Many women living with HIV have significant trauma exposure histories prior to, at the time of, and following diagnosis, and are more likely to encounter traumatic stressors than women in the general population [2]. Women living with HIV are also twice as likely than men living with HIV to develop posttraumatic stress disorder (PTSD) [3, 4], which has been attributed to women being more likely to experience gender-based stigma and violence, social exclusion, and traumatic life events [5]. Multiple authors attribute the development of PTSD among women living with HIV to traumatic events and circumstances that simultaneously contribute to their increased likelihood of acquiring HIV infection [6]. For example, exposure to abuse in childhood and adulthood can contribute to women’s subsequent participation in sexual and drug use behaviors that increases their risk for HIV acquisition [6]. Structural barriers, such as living in poverty, have also been posited as key contributors to increased exposure to traumatic stressors including domestic violence, crime, and other forms of assault and violence [7].

Women living with HIV experience significant stressors following an HIV diagnosis, including mental health difficulties, HIV-related stigma, and stressors due to limited social support networks, all of which are risk factors associated with the onset of PTSD when exposed [5]. Trauma and PTSD have negative impacts on health status (e.g., higher reported experiences of bodily pain, poorer perception of physical health, and poorer physical functioning) and contribute to increased health care utilization for people living with HIV [8]. It is also hypothesized that people living with HIV who are trauma survivors may face unique challenges related to antiretroviral medication adherence given the high co-morbidity between PTSD and depression and the association between PTSD and difficulties establishing trusting relationships and accessing social support, which are key determinants of antiretroviral adherence [6, 9]. Additionally, women living with HIV report high levels of perceived HIV-related stigma—awareness of negative social attitudes toward people living with HIV—and fears of mistreatment [10]. This may be potentially exacerbated by their experiences of trauma, which may impact whether or not they seek appropriate health care services, and access social supports [11]. Reducing the impact of traumatic stressors can enhance quality of life, prevent the development of more severe psychological symptoms, and facilitate effective coping of stigmatizing experiences [12].

A significant need has been identified for trauma-informed interventions for people living with HIV [2]. Trauma-informed refers to the incorporation of acknowledgment, awareness and sensitivity to the impact of trauma on peoples’ experiences into care [13, 14]. Studies to date have not examined posttraumatic stress symptoms (PTSS) in women living with HIV in Canada, nor have they described correlates of these outcomes among a large sample of women living with HIV in this context. There are context-specific variables associated with living with HIV that vary by region across Canada such as varying modes of transmission, experiences of HIV-related stigma and health care utilization; it is therefore important to examine the incidence as well as correlates of PTSS in Canada [15, 16, 17, 18]. Additionally, literature from other geographical areas suggests that there are often regional differences in the experiences of posttraumatic symptoms, associated with experiences of discrimination, social support and emotional symptoms, as well as social and historical context, drawing on social perceptions, connectedness and self-evaluation [19, 20]. Understanding the variables that are associated with posttraumatic symptoms including racism, social support, HIV stigma, HIV status disclosure, gender discrimination, resilience, and demographic variables can help inform interventions and better understand the scope of PTSS for women living with HIV in Canada. Given Canada’s vast size, regional differences, and the differing demographic characteristics of women living with HIV in each of these areas, understanding if PTSS symptoms vary by region is important to examine in order to facilitate appropriate care.

This study aims to examine rates of self-reported PTSS in women living with HIV in Canada, and to determine whether or not differences exist in the presence of PTSS based on three geographic locations: British Columbia, Ontario and Québec. Additionally, this study will examine the variables associated with PTSS in this sample, including demographic, social and interpersonal variables, and examine them using data-driven model building, given the exploratory nature of the analyses.

## Methods

### Study design and participants

This research was approved by the Women’s College Hospital Research Ethics Board (2011-0024-E), the harmonized University of British Columbia and Simon Fraser University Research Ethics Boards (H12-03326), and the McGill University Health Centre Research Ethics Board (2012–953 11–102). All clinical investigation was conducted according to the principles expressed in the Declaration of Helsinki. Written informed consent was obtained from all participants.

Participants were part of the Canadian HIV Women’s Sexual & Reproductive Health Cohort (CHIWOS) study, a multisite, prospective cohort study examining women-centered HIV care in Canada. CHIWOS is a community-based research project, guided by Social Determinants of Health and Critical Feminist frameworks [21], and adherent to meaningful involvement of people living with HIV (MIWA) principles. Full descriptions of the CHIWOS cohort, principles surrounding it, recruitment strategies, and involvement of Peer Research Associates (PRA) have been described elsewhere [22]. This analysis includes baseline cross-sectional data of participants from British Columbia, Ontario and Québec.

Non-random purposive sampling was used to recruit a geographically representative sample of women living with HIV in British Columbia, Ontario and Québec in order to replicate the geographic distribution of women living with HIV in these provinces [23, 24, 25]. Particular attention was made to recruit women in harder-to-reach contexts, such as Indigenous women (a term used to be reflective of First Nations, Inuit, Métis, and self-identified non-status Aboriginal women), women living in Northern Canada, trans-women and women not accessing health care. Participants were recruited via PRA networks, AIDS service organizations, community-based organizations, HIV clinics, online social media, peer referrals, and the networks of the CHIWOS Community Advisory Board and national steering committee. Responses were collected between April 2013 and April 2015.

### Procedures

PRAs administered an online questionnaire (using FluidSurveys software) to participants in either English or French in-person, over the phone, or over Skype. The questionnaire included questions regarding sociodemographic characteristics, health care and service utilization, sexual and reproductive health, HIV-related medical history, mental health, stigma, substance use, violence and abuse, resilience and quality of life. Participants received an honorarium of $50 for participating.

### Measures

Potential covariates of the primary outcome, posttraumatic stress symptoms, were determined a priori based on posttraumatic stress disorder literature and variables deemed by the advisory team as being relevant for women living with HIV. In addition to provincial location, sociodemographic and clinical variables were examined as potential covariates (see Table 1 for a full listing). Ethnicity was used as a descriptive variable, but was removed from further analyses as it shared considerable variance with immigration status and racism, and also theoretically made sense to remove, given the relationship with these other variables and PTSS.

**Table 1:** Descriptive characteristics.

	Total Sample N = 1424		British Columbia N = 356		Ontario N = 713		Québec N = 355	
Variable	N (Yes)	%	N (Yes)	%	N (Yes)	%	N (Yes)	%
Live in urban area	1171	82.2%	219	61.5%	654	91.7%	298	83.9%
Sex at birth								
Female	1367	96.0%	344	96.6%	684	95.9%	339	95.5%
Male	52	3.7%	10	2.8%	28	3.9%	14	3.9%
Intersex or other sex at birth	3	0.2%	0	0%	1	0.1%	2	0.6%
Gender identity								
Woman	1361	95.6%	342	96.1%	679	95.2%	340	95.8%
Transwoman	54	3.8%	11	3.1%	29	4.1%	14	3.9%
Two-Spirited/Gender Queer/Other	9	0.6%	3	0.8%	5	0.7%	1	0.3%
Sexual orientation								
LGBTTQ	180	12.6%	61	17.1%	92	12.9%	27	7.6%
Heterosexual	1239	87.0%	294	82.6%	617	86.5%	328	92.4%
Ethnicity								
Indigenous	318	22.3%	161	45.2%	149	20.9%	8	2.3%
Black	418	29.4%	28	7.9%	227	31.8%	163	45.9%
Caucasian	585	41.1%	139	39.0%	280	39.3%	166	46.8%
All other ethnicities	103	7.2%	28	7.9%	57	8.0%	18	5.1%
Immigration status								
Citizen or permanent resident	1322	92.8%	348	97.8%	655	91.9%	319	89.9%
Refugee or other immigration status	97	6.8%	7	2.0%	54	7.6%	36	10.1%
Food insecurity	908	63.8%	221	62.1%	478	67.0%	209	58.9%
Education								
Less than high school	227	15.9%	93	26.1%	81	11.4%	53	14.9%
High school or higher	1190	83.6%	260	73.0%	629	88.2%	301	84.8%
Household annual income								
Less than $20,000	903	63.4%	259	72.8%	425	59.6%	219	61.7%
$20,000 or higher	478	33.6%	86	24.2%	263	36.9%	129	36.3%
Stable housing	1272	89.3%	297	83.4%	629	88.2%	346	97.5%
Involvement in sex work	82	5.8%	36	10.1%	30	4.2%	16	4.5%
History of incarceration	524	36.8%	222	62.4%	205	28.8%	97	27.3%
Having attended a residential school								
Self	14	1.0%	11	3.1%	2	0.3%	1	0.3%
Family member	106	7.4%	81	22.8%	22	3.1%	3	0.8%
Ever having lived in a First Nations Community	130	9.1%	81	22.8%	45	6.3%	4	1.1%
Ever in the care of Child Protective Services	273	19.2%	113	31.7%	94	13.2%	66	18.6%
Ever been in foster care	282	19.8%	129	36.2%	98	13.7%	55	15.5%
Heavy or binge drinking	242	17.0%	85	23.9%	101	14.2%	56	15.8%
Cannabis use	362	25.4%	127	35.7%	178	25.0%	57	16.1%
Recreational drug use (past year)^a^	257	18.0%	127	35.7%	83	11.6%	47	13.2%
Injection drug use (past year)	123	8.6%	74	20.8%	33	4.6%	16	4.5%
Ever taken antiretroviral medication	1247	87.6%	340	95.5%	567	79.5%	340	95.8%
Ever experienced violence as an adult	1059	74.4%	316	88.8%	472	66.2%	271	76.3%
Ever experienced violence as a child	899	63.1%	270	75.8%	416	58.3%	213	60.0%
Suspected mode of HIV transmission								
Consensual sex	685	48.1%	113	31.7%	416	58.3%	156	43.9%
Non-consensual sex	219	15.4%	61	17.1%	94	13.2%	64	18.0%
Sharing needles	262	18.4%	127	35.7%	74	10.4%	61	17.2%
Blood	70	4.9%	15	4.2%	30	4.2%	25	7.0%
Perinatal	50	3.5%	6	1.7%	30	4.2%	14	3.9%
Contaminated needles	17	1.2%	12	3.4%	2	0.3%	3	0.8%
Other	5	0.4%	3	0.8%	1	0.1%	1	0.3%
Variable	Mean (SD)	Range	Mean (SD)	Range	Mean (SD)	Range	Mean (SD)	Range
Age	42.83 (10.62)	16–74	43.80 (10.46)	19–72	41.06 (10.41)	16–73	45.40 (10.64)	18–74
Length of time in Canada (years)^b^	13.74 (12.73)	0–62	21.71 (17.31)	1–57	12.50 (11.91)	0–62	13.13 (11.39)	1–46
Posttraumatic stress symptoms	14.17 (6.68)	6–30	15.33 (6.43)	6–30	13.26 (6.60)	6–30	14.79 (6.85)	6–30
Depressive symptoms	10.03 (7.56)	0–30	11.76 (7.42)	0–30	9.22 (7.55)	0–30	9.91 (7.44)	0–30
HIV-related stigma	57.23 (20.00)	0–100	57.16 (20.27)	0–100	59.87 (19.90)	0–100	51.97 (18.92)	0–100
Disclosure	8.24 (4.97)	0–20	9.70 (5.11)	0–20	8.01 (4.62)	0–20	7.22 (5.19)	0–20
Social support	14.14 (4.44)	4–20	14.18 (4.38)	4–20	14.52 (4.54)	4–20	13.30 (4.21)	4–20
Resilience	62.17 (8.07)	18–70	59.97 (9.27)	19–70	62.31 (7.85)	32–70	64.10 (6.55)	32–70
Racism	19.06 (11.06)	8–48	21.26 (11.22)	8–48	20.00 (11.31)	8–48	14.87 (9.15)	8–48
Gender discrimination	19.59 (10.08)	8–48	21.74 (9.88)	8–48	20.56 (10.37)	8–48	15.45 (8.37)	8–48

Note. LGBTTQ = lesbian, gay, bisexual, queer, transgender, Two-Spirited, queer.

^a^Excluding cannabis and injection drugs

^b^n = 500, as only included for those not born in Canada

Additionally, the following scales were used

#### Posttraumatic stress symptoms (PTSS). (abbreviated PTSD checklist, PCL) [26]

The abbreviated, 6-item PCL is a self-report measure used to screen for PTSD. The measure uses a five-point Likert scale ranging from 1 (not at all) to 5 (extremely), with possible scores ranging from 6 to 30. As a screening metric, a score of ≥14 is considered positive and has sensitivity of .80 and specificity of .76 for PTSD [18]. In the current study, the measure had a Cronbach’s alpha of .91.

#### Depressive symptoms. (Center for Epidemiological Studies Depression Scale, CES-D 10) [27]

This 10-item version of the CES-D is rated on a four-point Likert scale from 0 (rarely or none of the time) to 3 (most or all of the time), with possible scores ranging from 0 to 30. The 10-item version has been validated among people living with HIV in British Columbia [28], and in the current study had a Cronbach’s alpha of .88.

#### HIV stigma. (HIV Stigma Scale– 10 item) [29]

For this study, a mid-point response of “neither agree nor disagree” was added to the scale. This edit was made to allow for respondents to not be placed in a “forced choice” position, per advice and direction from the community advisory team. The measure is on a five-point Likert scale ranging from 0 (strongly disagree) to 4 (strongly agree), multiplied by 2.5 to give a total score out of 100. The measure had a Cronbach’s alpha of .85 in the current study.

#### Disclosure. (HIV/AIDS Quality of Life–Disclosure subscale) [30]

This subscale assesses the extent to which individuals living with HIV are able to share or disclose about their lives with others. This subscale consists of five items on a five-point Likert scale ranging from 0 (strongly disagree) to 4 (strongly agree). The scale anchors “none of the time” to “all of the time” were edited for this questionnaire to better reflect a general report as opposed to within a timeframe. The internal consistency in the current study is Cronbach’s alpha = .83.

#### Social Support. (Abbreviated Medical Outcomes Study Social Support scale, MOS-SSS) [31]

The four-item version of the MOS-SSS assesses presence of social support, and is on a five-point Likert scale from 1 (none of the time) to 5 (all of the time), with possible scores ranging from 4 to 20. In the current study, the measure has Cronbach’s alpha of .85.

#### Resilience. (Resilience Scale) [32]

The 10-item version of the Resilience Scale was used, including items such as “When I’m in a difficult situation, I can usually find my way out of it.” The measure is scored on a 7-point Likert scale from disagree to agree, with possible scores ranging from 10 to 70. Cronbach’s alpha in the current study is .91.

#### Racism. (Everyday Discrimination Scale–Racism subscale) [33, 34]

The eight-item measure, which was edited to allow for trans-inclusive language for this study, uses a six-point Likert scale, and was scored from 1 (never) to 6 (almost every day), with possible scores ranging from 8 to 48. In this study, Cronbach’s alpha was .96.

#### Gender discrimination. (Everyday Discrimination Scale—Gender discrimination subscale) [33]

The eight-item measure, which was edited to allow for trans-inclusive language for this study, uses a six-point Likert scale, and was scored from 1 (never) to 6 (almost every day), with possible scores ranging from 8 to 48. In this study, Cronbach’s alpha was .94.

### Data analysis

All statistical analyses were conducted using IBM SPSS Statistics 22.0. Missing data were excluded using list-wise deletion unless a mean score was able to replace a missing value (this was applied when 80% of data was available for a measure). Incidence of PTSD was determined as a percentage of individuals who scored 14 or higher on the PCL [26], and descriptive statistics were examined for first the whole sample, and then by province after determining that PTSD incidence varied by region (established by ANOVA and Tukey’s HSD post-hoc comparisons). Given that PTSD incidence varied by province, all subsequent analyses were conducted separately by province. Subsequently, PTSS as a linear variable was used for the regression analyses. Backward and forward linear regression models were conducted for each of the provinces with all variables (all measures listed, as well as all clinical and sociodemographic variables in Table 1). Backward and forward linear regression models allow for a convergence of information for the statistically significant variables contributing to the regression model. Specifically, if a variable is a statistically significant predictor in both the backward and forward regression models (as a conservative method of determining inclusion), it was included in the final multivariable analysis using PTSS as the outcome variable for the province. The multivariable regression models are presented as the final regression models. Bivariate correlations were conducted for all included final model predictor variables with PTSS. Multicollinearity was not detected as calculated variance inflation factor (VIF) scores were below 10 and all standard error scores were below two [35].

## Results

A total of 1424 women are included in the cohort, and 1405 had complete data for the primary outcome variable, posttraumatic stress symptoms. Three hundred and fifty-six women were in British Columbia, 714 in Ontario and 355 in Québec. Sociodemographic characteristics, as well as mean scores for variables of interest, can be found in Table 1. The incidence of PTSS scores consistent with a cut-off for PTSD (a score of ≥14) was 47.1% in the whole sample, and 55.9% in British Columbia, 39.1% in Ontario, and 54.1% in Québec. There was a significant difference among the provinces on PTSS scores, *F*(2, 1402) = 13.53, *p* < .001. Post-hoc multivariable comparisons revealed that scores differed between Ontario and both Québec (*p* = .001) and British Columbia (*p* < .001), but not between British Columbia and Québec (*p* = .53).

### Differing provincial posttraumatic stress disorder regression models

The regression model for British Columbia included: depressive symptoms, gender discrimination, ever having taken antiretroviral medication (negatively associated), current injection drug use (negatively associated), and resilience (negatively associated), and accounted for 59% of the variance in the PTSS score. All variables were significant independent predictors of posttraumatic stress symptoms. The full model can be seen in Table 2.

**Table 2:** Multivariable regression model of predictors of posttraumatic stress symptoms in British Columbia (n = 341).

	*B (CI)*	*SE B*	B	*p*
Constant	16.30 (12.04, 20.56)	2.17		< .001
Ever on ART*	-5.90 (-8.07, -3.72)	1.10	-.19	< .001
Current IDU*	-1.37 (-2.52, -0.23)	0.58	-.09	.019
Depressive symptoms	0.53 (0.46, 0.60)	0.04	.61	< .001
Resilience	-0.07 (-0.13, -0.02)	0.03	-.10	.013
Gender discrimination	0.14 (0.09, 0.18)	0.02	.21	< .001

Note. *R*^2^ = .59, *p* < .001. CI = 95% confidence interval, SE = standard error, ART = antiretroviral treatment, IDU = injection drug use.

*0 = no, 1 = yes.

The n reported in this table is lower than the total n for the province due to missing data on one or more of the predictor variables in the regression model.

The regression model for Ontario included: sexual orientation, immigration status, ever under the care of Child Protective Services, ever having experienced violence as an adult, self-reported mode of HIV transmission (non-consensual sex and sharing needles), HIV stigma, depressive symptoms, gender discrimination, social support (negatively associated), and resilience (negatively associated), and accounted for 66% of the variance in PTSS score. Sexual orientation, experiences of violence as an adult, and resilience were non-significant predictors and were removed for model parsimony. The final model accounted for 64% of the variance in PTSS score, and all variables were significant independent predictors (Table 3).

**Table 3:** Multivariable regression model of predictors of posttraumatic stress symptoms in Ontario (n = 593).

	*B (CI)*	*SE B*	B	*p*
Constant	7.32 (5.22, 9.41)	1.07		< .001
Immigration status*	-1.65 (-3.01, -0.29)	0.69	-.06	.018
Ever in the care of CPS**	1.20 (0.28, 2.12)	0.47	.07	.011
Non-consensual sex as mode of transmission**	1.36 (0.41, 2.30)	0.48	.07	.005
HIV stigma	0.02 (0.00, 0.04)	0.01	.07	.014
Depressive symptoms	0.56 (0.51, 0.62)	0.03	.65	< .001
Social support	-0.09 (-0.18, -0.01)	0.04	-.06	.037
Gender discrimination	0.09 (0.05, 0.12)	0.02	.14	< .001

Note. *R*^2^ = .64, *p* < .001. CI = 95% confidence interval, SE = standard error, CPS = Child Protective Services.

*0 = refugee, other immigration status, 1 = citizen, permanent resident.

**0 = no, 1 = yes.

The n reported in this table is lower than the total n for the province due to missing data on one or more of the predictor variables in the regression model.

The regression model for Québec included: ever having experienced violence as a child, incarceration, food insecurity, cannabis use, current recreational drug use (negatively associated), depressive symptoms, HIV stigma, disclosure (negatively associated), gender discrimination, resilience (negatively associated) and self-reported mode of HIV transmission (consensual sex, negatively associated), and accounted for 59% of the variance in PTSS score. Having experienced violence as a child, cannabis use, current recreational drug use, consensual sex, HIV stigma, gender discrimination and resilience were non-significant predictors and were removed for model parsimony. The final model accounted for 54% of the variance in PTSS score, and all variables were significant independent predictors (Table 4).

**Table 4:** Multivariable regression model of predictors of posttraumatic stress symptoms in Québec (n = 327).

	*B (CI)*	*SE B*	B	*p*
Constant	9.35 (8.09, 10.62)	0.64		< .001
Incarceration	1.89 (0.65, 3.12)	0.63	.12	.003
Food insecurity	2.43 (1.38, 3.48)	0.54	.17	< .001
Depressive symptoms	0.57 (0.49, 0.64)	0.04	.61	< .001
Disclosure	-0.29 (-0.39, -0.18)	0.05	-.21	< .001

Note. *R*^2^ = .54, *p* < .001. CI = 95% confidence interval, SE = standard error.

The n reported in this table is lower than the total n for the province due to missing data on one or more of the predictor variables in the regression model.

Correlations of the variables included in the final regression models are seen in Table 5.

**Table 5:** Correlations of variables in final regression models with posttraumatic stress symptoms.

	PTSS Full Sample	PTSS British Columbia	PTSS Ontario	PTSS Québec
Ever on ART^a^	.08*	-.22**	.12*	.07
Current IDU^a^	.14**	.07	.17**	.18**
Immigration status^b^	-.10**	.05	-.17**	-.07
Ever in the care of CPS^a^	.13**	.05	.15**	.12*
History of incarceration	.15**	.04	.14**	.19**
Food insecurity	.21**	.19**	.20**	.30**
Depressive Symptoms	.73**	.71**	.76**	.69**
HIV-related stigma	.33**	.37**	.30**	.42**
Disclosure	-.22**	-.22**	-.25**	-.22**
Social support	-.38**	-.26**	-.50**	-.21**
Resilience	-.42**	-.39**	-.47**	-.37**
Gender discrimination	.34**	.38**	.35**	.36**
Consensual sex as mode of transmission^a^	-.22**	-.08	-.26**	-.14*

Note. PTSS = Posttraumatic stress symptoms, ART = antiretroviral treatment, IDU = injection drug use, CPS = Child Protection Services.

^a^0 = no, 1 = yes.

^b^0 = refugee, other immigration status, 1 = citizen, permanent resident.

*p < .005

** *p* < .001.

## Discussion

Women living with HIV in Canada are experiencing high levels of posttraumatic stress symptoms. The incidence of PTSS scores consistent with PTSD in our sample was much higher than the prevalence of PTSD in the general population at 7 to 10%; at 47.1% incidence for our entire sample, with some difference across regions, our findings are consistent with previous research [4]. Because of the pernicious nature of these symptoms and their ramifications on physical health, relationships, and quality of life, trauma-informed care and interventions are clearly needed.

The regression models in each province account for a large amount of the variance (59%, 64%, and 54% of the variance in British Columbia, Ontario, and Québec, respectively), suggesting the variables included in the analyses offer a relatively comprehensive picture of contributors to PTSS. Separate models for each province suggest that the correlates and predictors of PTSS may be geographically different, supported by the contextually different experience of living with HIV in these regions. In British Columbia never having been on antiretroviral medication and less current injection drug use are significant predictors of PTSS. These variables represent a context that is more likely to have trauma exposure and perhaps less access or incentive to receive care. Notably, current injection drug use is negatively associated with PTSS, indicating that it may be being used as a coping mechanism, acting as a mediator, or masking the experience of symptoms [36, 37]. The relationship between, directionality, and reciprocity of injection drug use and PTSS should be investigated in future studies.

In Ontario, immigration status (specifically, having refugee or other immigration status, as opposed to being a citizen or permanent resident) was a predictor of PTSS, reflecting the demographic make-up of women living with HIV in Ontario, and the intersecting stressful experience of having uncertain immigration status. Additionally, refugee status can inherently suggest exposure to traumatic events, which can be associated with PTSS. While the percentage of women with refugee or other immigration status was higher in Québec, it is possible that the context in Ontario, for example women’s experiences prior to and after immigration, including access to secure housing and other facets of safety and security, may vary across region, and warrants further investigation. Acquiring HIV via forced sex was a predictor of PTSS in Ontario. This could be construed as a traumatic event in and of itself, along with involvement with Child Protective Services (also a significant predictor).

In Québec, lower ability to disclose information about HIV diagnosis was a predictor of PTSS, and speaks to the importance of perceived positive social support regarding disclosure, and the ability to harness that support. A history of incarceration and a lack of food security were also predictors of PTSS, highlighting the stressors associated with involvement in the legal system, which can occur pre, during and post incarceration, as well as with the ongoing stress associated with unstable access to food and resources.

Across provinces we see that depressive symptoms are a significant predictor of PTSS. Depression and PTSD are highly correlated in the literature [38], and these findings support that association. In Ontario and British Columbia, we also see that gender discrimination is a significant predictor of PTSS. The stress associated with gender discrimination, as well as with HIV-related stigma (seen as a significant predictor in Ontario) may create a context that can exacerbate PTSS, and may impact coping around traumatic and stressful situations. These findings corroborate prior research with women living with HIV in Ontario that reported associations between depression, poorer wellbeing, and reduced quality of life with factors including HIV-related stigma, gender discrimination, lower coping and lower resilience [39, 40]. Resilience and social support, in British Columbia and Ontario, respectively, are negatively associated with PTSS, demonstrating the bolstering effect of these two constructs. They may be an area for targeted intervention in the future to help decrease PTSS. Additionally, these results corroborate findings that posttraumatic reactions can vary geographically, and therefore regional differences may be important to consider in terms of both HIV care and addressing posttraumatic reactions [17, 20].

Given the sociodemographic characteristics of our participants, and the PTSS literature, the high rates of PTSS in our sample are not entirely surprising. Compared to their representation in the general population, Indigenous and African, Caribbean and Black (ACB) women are disproportionately represented in the HIV epidemic in Canada; and women from these ethnicities accounted for a significant proportion of our sample [41, 42]. Indigenous women are more likely to have encountered traumatic life events because of Canada’s history of colonization, the residential school system, and intergenerational trauma [43]. ACB women in our study, many of whom were not born in Canada, may have been exposed to civil unrest and conflict in their countries of origin, further contributing to their likelihood of increased PTSS. A significant proportion of participants across all regions identified one or more factors that can contribute to PTSS including living in poverty (63% were living on less than $20,000 per year; 63.8% identified food insecurity), a history of incarceration (37%), involvement with the child welfare system (19.2% had ever been in the care of a child protection service, 19.8% ever in foster care), and a history of violence (63.1% experienced violence as a child, 74.4% experienced violence as an adult). Taken together, these findings support the need for trauma-informed care and interventions that address both women’s structural and psychosocial needs.

Limitations of the study include using self-report measures and the abbreviated PCL. Self-report measures may create response bias, and therefore corroboration with clinician-administered measures, particularly of psychiatric symptoms. While using the full version and clinician-administered measures would give a clearer picture if PTSD is indeed present in its full diagnostic form, the ability to ascertain this data on PTSS in a low-burden manner from a very large sample is desirable in terms of getting a representative picture of the general trends of PTSS in these three provinces. Additionally, it is unknown what traumatic event(s) women are reporting their symptoms based on, or indeed the nature and number of traumatic events in their lives, and how proximal the event(s) they are referencing are to the present day. Future directions could include using a life events checklist (to determine type of traumatic event experienced), as well as assessing the course of PTSS longitudinally to determine the course and trajectory of PTSS over time for this group.

Further assessment of psychiatric symptoms, including measures of generalized anxiety, social anxiety, postpartum depression, and insomnia, for example, along with information regarding ongoing, peritraumatic event experiences and ongoing implications from traumatic events may help contribute to understanding PTSS symptoms more fully, and may account for some the variance in the regression models as yet unaccounted for. Finally, participants were offered the opportunity to skip out of any section category they did not feel comfortable answering and 107 participants from Ontario had missing data for at least one variable included in the model. The most frequent missing data were encountered for involvement in sex work, experiences of violence and suspected mode of HIV transmission. There was no significant difference on PTSS between individuals who did and did not have missing data for this analysis. Given the sensitive nature of these questions, missing data may be more common among participants with involvement in sex work, experiences of violence and non-consensual sex as an HIV transmission mode. This may lead to an underestimation of the association between PTSS and non-consensual sex which was found to be significant in the Ontario data. It may also have impacted model building and more complete data on experiences of violence and involvement in sex work may have led to their inclusion as significant covariates. This is a limitation of the data and alternate methods for data collection on these sensitive topics may be needed. Implementing and testing trauma-informed care and trauma-focused interventions for women living with HIV, and testing these by region, would both address the needs of the women and provide further information about geographical and covariate differences.

In conclusion, these results demonstrate that women living with HIV across Canada are significantly affected by posttraumatic stress symptoms, and that every attempt should be made to support trauma-informed practices, and access to trauma-focused interventions should PTSD be present. Bolstering resilience and social support may be a concrete next step in terms of supporting women living with HIV who are experiencing PTSS.
